# Perception and experience of obstetric violence in postpartum women at a public hospital in Peru: a mixed study

**DOI:** 10.17843/rpmesp.2025.421.14281

**Published:** 2025-03-11

**Authors:** Paola M. Marcos-Garces, Stefanny M. Moran-Ruiz, Yuly R. Santos-Rosales, Miriam Y. Correa-Lopez

**Affiliations:** 1 Professional School of Obstetrics, Faculty of Medicine, National University of San Marcos, Lima, Peru. National University of San Marcos Professional School of Obstetrics, Faculty of Medicine National University of San Marcos Lima Peru

**Keywords:** Obstetric violence, Violence against women, Gender violence, Humanized birth, Obstetric birth

## Abstract

**Objectives.:**

To determine the perception and experience of obstetric violence during childbirth among postpartum women in a public hospital in Peru.

**Materials and methods.:**

Mixed, descriptive and cross-sectional study during the quantitative phase and with a phenomenological design during the qualitative phase. The population was 444 postpartum women, with a sample made up of 139 postpartum women who had vaginal delivery (surveys) and 21 postpartum women (semi-structured interviews). The study was carried out during the months of April to December 2023.

**Results.:**

We identified that 25.2% of the surveyed women perceived obstetric violence during childbirth and the postpartum period; however, when asked about specific forms of obstetric violence, 100% of postpartum women reported having experienced some form of physical violence and 97.8% responded that they experienced some form of psychological obstetric violence and all the participants reported having suffered at least one form of obstetric violence. Regarding psychological violence, of the total number of puerperal women, 69.1% perceived that they were not informed about consent before signing and undergoing an intervention, 53.2% stated that the staff that assisted them during delivery did not identify themselves by name or profession. With regard to physical obstetric violence, 96.4% did not have a trusted person present during childbirth, 91.4% did not have the option to choose the position in which to give birth (horizontal or vertical), and 76.3% did not have the time for skin-to-skin contact with their newborn. Obstetric violence is expressed in feelings of fear, anguish, anxiety, frustration and loneliness, which puts maternal and neonatal health at risk.

**Conclusions.:**

postpartum women perceive obstetric violence on a psychological level due to the way they are treated by healthcare professionals and on a physical level due to the practices carried out during childbirth, which negatively affect their experiences, causing an emotional impact.

## INTRODUCTION

Childbirth is a physiological process that culminates in the delivery of the conceived product ^1^. However, medicalization and the use of technology have transformed humanized childbirth by prioritizing medical interventions, reducing women’s autonomy, and depersonalizing the birth experience ^2^^,^^3^. This has created tension between respect for natural processes and excessive medical intervention, affecting the birth experience. The World Health Organization (WHO) recognizes women’s right to dignified care, free from violence, but there are reports of incidents that generate mistrust and rejection of health services ^4^. It is therefore essential to provide quality care that respects women’s autonomy through practices such as birth accompaniment by someone of their choice, effective communication between doctor and patient, and the promotion of mobility, choice of positions, access to fluids, and pain relief ^1^. These actions are aligned with the third Sustainable Development Goal, which seeks to promote health and well-being at all stages of life ^5^^,^^6^.

Worldwide, there is no consensus on the definition of obstetric violence ^7^^-^^10^. In Peru, this form of violence is recognized as a type of gender-based violence ^11^. According to the “National Plan Against Gender Violence 2016-2021,” it is defined as “all acts of violence by health personnel related to reproductive processes, which are expressed in dehumanizing treatment, abuse of medicalization, and pathologization of natural processes” ^12^^,^^13^.

Obstetric violence manifests itself in various situations, such as the failure of health personnel to obtain informed consent (73.6%), disregard for the patient’s opinion and decision, lack of information about vaginal examinations (34.8%) ^14^, failure of health personnel to identify themselves by name (32.8%), denial of accompaniment during childbirth (90.4%), inappropriate administration of medication (99.6%) ^13^, or the performance of procedures by students without the patient’s consent ^3^^,^^12^^,^^13^. These practices reflect the violation of women’s reproductive rights, causing mistrust of health services and perpetuating structural violence in obstetric care ^15^^,^^16^.

Obstetric violence is a problem that requires urgent attention due to its negative effects ^17^ on women’s lives, on timely access to healthcare, and because it violates their reproductive rights. In this context, this study aimed to determine the perception of obstetric violence during childbirth among postpartum women in a public hospital in Peru in 2023.

**KEY MESSAGES**

Motivation for the study. There are gaps in humane care during childbirth, where obstetric violence is evident, and it is important to understand the perceptions of postpartum women regarding this type of violence.

Main findings. We found that 25.2% of the participants reported having experienced obstetric violence; however, all participants experienced some form of obstetric violence, such as not receiving an explanation about informed consent for procedures, health personnel not identifying themselves, and not having the option to choose the position and accompaniment during childbirth.

Implications. There is an urgent need to empower pregnant women about the importance of exercising their reproductive rights and identifying obstetric violence in order to implement recommendations and conditions for a humanized birth.

## MATERIALS AND METHODS

### Study design and sample

Mixed-method study ^18^ with a concurrent triangulation design, which allowed for the simultaneous collection and analysis of quantitative and qualitative data, to be integrated later in the interpretation phase. The quantitative approach used a descriptive cross-sectional design, while the qualitative approach followed a phenomenological design, focusing on describing and understanding the participants’ experiences of obstetric violence. This approach provided a comprehensive view of the perceptions and manifestations of obstetric violence, contrasting numerical evidence with the subjective accounts of postpartum women.

The sample consisted of 139 participants and Fisher’s formula was used to extract a representative sample. N: universe or population 444, p: probability in favor 0.5, q: probability against 0.5, n: sample size 139, e: estimation error 0.03, alpha: confidence level coefficient 1.960.

For the quantitative approach, the estimate from the Daniel Alcides Carrión National Hospital was 444 postpartum women (according to 2022 records), and the sample consisted of 139 postpartum women selected by simple random sampling. For the qualitative approach, we included 21 postpartum women selected by theoretical saturation criteria.

The selection criteria for the population were: all women who had given birth vaginally at the Daniel Alcides Carrión National Hospital in the Callao region of Peru between April and December 2023 (quantitative) and, for the qualitative approach, women who answered yes to the questions on obstetric violence. Likewise, for both approaches, women of foreign nationality, who spoke a language other than Spanish, who had special needs, or who were admitted to the Intensive Care Unit, and those who did not sign informed consent and/or assent were excluded.

### Variables, instrument, and data collection

For the quantitative approach, obstetric violence (OV) was assessed in two ways: one through a direct question to explore whether they perceived having suffered obstetric violence, and the other through specific experiences of OV in two dimensions, physical and psychological violence. The pregnant woman was considered to have experienced OV when she presented at least one physical or psychological manifestation ^19^. This definition and classification took into account the WHO ^2^, the Ombudsman’s Office ^11^, and the National Plan on Gender Violence ^12^.

It was categorized as psychological violence when there was a positive response to the following manifestations: derogatory, discriminatory, ironic, and disparaging expressions; scolding for expressing pain; omission of informed consent, among others ^3^^,^^12^. Physical violence was considered to be a positive response to any of the following manifestations: unjustified vaginal touching, Kristeller or Hamilton maneuvers, medicalization during childbirth, artificial rupture of membranes, early clamping of the umbilical cord, unjustified denial of mobility during childbirth, inability to choose the position of childbirth, presence of people unrelated to the delivery room without the mother’s consent, among others) ^3^^,^^20^. For the qualitative phase, the main category was the perception of OV, and two subcategories were considered: meaning and emotions generated by physical obstetric violence and meaning and emotions generated by psychological obstetric violence.

We used a survey for the quantitative approach, this instrument was adapted from Adjunct Report No. 023-2020-DP/ADM ^12^, from the survey in the article Obstetric violence in Chile: women’s perceptions and differences between health centers ^19^, from the survey in the article Obstetric violence in Spain: reality or myth? 17,000 women give their opinion ^20^. The questionnaire consisted of 38 questions structured in three blocks; it was validated by expert peers, has a V of Aiken validity of (0.96) and a reliability of 0.77 (Kunder Richardson). Block I on psychological violence has 16 questions, block II on physical violence has 17 questions, and block III on information on obstetric violence has 5 questions. Postpartum women were selected through simple random sampling (lottery).

For the qualitative approach we used interviews; the instrument was a semi-structured interview guide with 18 questions divided into two blocks: Block I on psychological violence with seven questions, and Block II on physical violence with 11 questions. The content of the interview guide was validated by expert peers, who reviewed the content to ensure the clarity and relevance of the questions (V AIKEN validity of 0.90). The interviews were conducted in distraction-free spaces, ensuring confidentiality, with an average duration of 20 minutes. They were recorded with prior consent and informed assent for independent minors, and then transcribed and analyzed.

### Analysis

**Figure 1:**
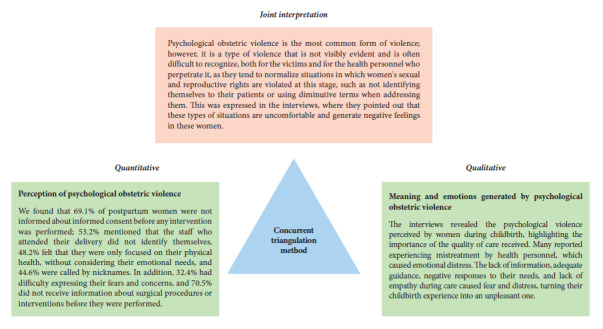
Schematic representation of quantitative and qualitative data triangulation for psychological obstetric violence.

Data were processed and analyzed using SPSS statistical software version v.29 for the quantitative analysis. The frequency and percentage of the variables were calculated. Data were organized by transcribing the interviews for the qualitative analysis. This was followed by coding, in which words and segments of data relevant to the study were identified, labeled, and grouped in order to explore ideas or patterns that were repeated among the interviewees. Next, categorization was carried out, identifying similarities and differences between the coded units in order to group them into thematic categories, which for the purposes of the study were the different types of obstetric violence experienced by the participants. These were described in a categorization matrix, and the results were then interpreted. To integrate quantitative and qualitative results, we applied the concurrent or convergent triangulation method ^18^, which was based on the simultaneous collection and analysis of quantitative and qualitative data, which were then integrated based on points of convergence and complementarity in the findings. These points of convergence were physical obstetric violence and psychological obstetric violence at the level of perceptions and experiences, which contributed to a joint and comprehensive interpretation of the phenomenon studied (obstetric violence) (Figure 1).

### Ethical considerations

This study was approved by the Ethics Committee of the Daniel Alcides Carrión National Hospital (No. 015 - 2023). Prior to data collection, participants were informed of the purpose of the study and the voluntary nature of their participation through informed consent and assent for minors who were independent and informed consent was obtained from their parents or guardians for those who were not ^21^. Data confidentiality was ensured through anonymity and its exclusive use for research purposes was stated.

## RESULTS

For interpretation purposes, the dimensions considered for the quantitative approach to population characterization were physical and psychological obstetric violence. For the qualitative approach, the categories were the experience of childbirth and the postpartum period, and the meaning and emotions generated by physical obstetric violence and psychological obstetric violence.

### Quantitative: Characterization of the study population

Most postpartum women (72%) were between 20 and 34 years old, with 18% between 14 and 19 years old; 69% were cohabiting, 70.5% had secondary education, and 71.2% were housewives (Table 1). Obstetric violence was initially addressed through a direct question, finding that 25.2% of the surveyed women perceived that they had suffered some type of obstetric violence during childbirth and the postpartum period. However, when asked about specific manifestations of OV, 100% of women reported having experienced some type of OV, 100% reported obstetric physical violence, and 97.8% reported psychological violence. These results indicate that, although obstetric violence is widely experienced, only a quarter of postpartum women recognize it as such (Table 2).

**Table 1:** General characteristics of postpartum women who participated in the study at a public hospital in Lima, Peru, 2023.

General characteristics (n=139)		n	(%)
Age			
	14 -19 years	25	(18.0)
	20-34 years	100	(72.0)
	>34 years	14	(10.0)
Marital status			
	Single	39	(28.1)
	Married	4	(2.9)
	Cohabiting	96	(69.0)
Education level			
	Primary school	5	(3.6)
	Secondary school	98	(70.5)
	Higher technical education	28	(20.1)
	University	8	(5.8)
Employment			
	Housewife	99	(71.2)
	Student	9	(6.5)
	Employed worker	7	(5.0)
	Self-employed worker	24	(17.3)

**Table 2:** Proportion of postpartum women who experienced obstetric violence in a public hospital in Peru, 2023.

Characteristics (n=139)	n	%
Experienced at least one instance of obstetric violence (physical or psychological)	139	100
Experienced physical obstetric violence	139	100
Experienced psychological obstetric violence	136	97,8
Experienced both types of obstetric violence	136	97,8

### Qualitative: Experiences during childbirth and the postpartum period

In the qualitative component, a common finding among the interviewees was the diversity of experiences during childbirth. For most, it was a pleasant experience due to the excitement of having their children, while for others it was uncomfortable and painful, either due to the physiological nature of childbirth or situations perceived as mistreatment by health personnel. Among these, the lack of adequate information and guidance on the procedures to follow stood out, which led to delays in care and unnecessary transfers between facilities, increasing the risk of complications and births outside the appropriate place or without the appropriate medical personnel.

“*I was 2 cm dilated and they told me to come back in 5 hours [...] When I arrived at the health center at 10 p.m., they examined me again and told me I was 5 cm dilated, but they said they couldn’t treat me there and that I should go to the hospital [...] So they refused to treat me, and I had to come on my own at 11 p.m. There were no taxis, I arrived at 8 cm dilated. When I got to the emergency room in Carrión, I asked for a chair and they told me there were no chairs. They made me walk, and when I got there, I told them I was already dilated, and they asked me for my referral. I explained that I didn't have one because at the clinic they told me to go to the hospital and that they would take care of me there. They accepted me, sat me down, and examined me. I was at 10 [...] Everything happened very quickly. They moved me from bed to bed. I was holding back the contractions and screaming in pain, and they told me not to scream. When I got here, I was moved to two more stretchers. They treated me with the same gown I was wearing because I was already at 10. They told me to close my legs because the baby was coming out [...]*”. 21-year-old postpartum woman.

Integration: Women who have experienced obstetric violence during childbirth and the postpartum period often present different characteristics than those who have not, which could become a risk factor for developing it, depending on the context and social, cultural, and economic conditions. Furthermore, it is important to consider that the meaning of obstetric violence varies according to the characteristics, conditions, and contexts in which it is experienced, which influences the perception and impact of this experience.

### Quantitative: Perceptions of physical obstetric violence

Among the most frequent manifestations of obstetric violence, 96.4% of women reported that they were not allowed to have a trusted person present during the entire delivery process, 91.4% did not have the option of choosing the position in which to give birth, and 76.3% reported that skin-to-skin contact with their baby was not respected. In addition, 71.2% experienced a delayed start to breastfeeding, 63.6% had their membranes artificially ruptured without prior communication, and 47.5% underwent repeated vaginal examinations by more than one person. Furthermore, 36.7% allowed other people to be present during their care without consent, 32.4% had early clamping of the umbilical cord, and 23% received medication without their knowledge or prior explanation. Other manifestations of obstetric violence were reported by less than 20% of participants (Table 3).

**Table 3:** Manifestations of physical obstetric violence in postpartum women from a public hospital in Peru, 2023.

Indicators	Yes		No	
	n	(%)	n	(%)
Repeated vaginal touching by more than one person at a given time	66	(47.5)	73	(52.5)
Maneuvers such as Kristeller, Hamilton maneuver, etc.	22	(15.8)	117	(84.2)
Surgical procedures (episiotomy, episiorrhaphy)	98	(70.5)	41	(29.5)
Administration of medication without knowledge or explanation of its importance or effects (oxytocin, hyoscine, dimenhydrinate, etc.)	32	(23.0)	107	(77.0)
Artificial rupture of membranes	88	(63.6)	51	(36.7)
Early umbilical cord clamping	45	(32.4)	94	(67.6)
Restricted mobility or inability to find a comfortable position before giving birth	25	(18.0)	114	(82.0)
Not having the option to choose the position in which to give birth (horizontal or vertical)	127	(91.4)	12	(8.6)
Not allowing the presence of a trusted person of choice during the entire birthing process	134	(96.4)	5	(3.6)
Did not place the baby in skin-to-skin contact immediately, even if he or she was born healthy.	5	(3.6)	134	(96.4)
The time for mother-child skin-to-skin contact was not respected.	106	(76.3)	33	(23.7)
Late initiation of breastfeeding	99	(71.2)	40	(28.8)
Allow other people to be present during the assessment or care without consent (trainees, interns, etc.)	51	(36.7)	88	(63.3)
After delivery, the staff inserted their entire hand to remove the remains of the placenta.	96	(69.1)	43	(30.9)
No medication was administered to reduce pain when they performed an examination with instruments to assess the uterus because there were remains of the placenta.	104	(74.8)	35	(25.2)
They inserted a finger into the anus without authorization or prior explanation.	6	(4.3)	133	(95.7)
They took photographs of the birth process without consent.	7	(5.0)	132	(95.0)

### Qualitative: Meaning and emotions generated by physical obstetric violence

Regarding the qualitative results related to physical obstetric violence, we highlight the importance of the procedures used to monitor the progress of pregnant women during childbirth. Many women expressed feeling uncomfortable because, although they understand that certain procedures are necessary, they were not informed or consulted to obtain their consent. In addition, the repetition of these procedures without explanation and the intervention of several health professionals, despite their discomfort and unease, made them feel vulnerable and emotionally affected.

*“Yes, I told them it made me uncomfortable, and they said, 'But we’ve given you anesthesia (analgesia), it can't hurt.’ […] Well, that’s also quite uncomfortable because every five minutes I think they put their hand in, and then another one came, and another one came, and they kind of touched you and pulled you, I don't know what, and the pain got worse, and you told them no, but they kept going. He just did it, but then he left and another doctor came and put his hand inside you.”* 16-year-old postpartum woman

Meeting the needs of patients in health facilities is a key indicator of quality, linked to the level of satisfaction and well-being of users. In this regard, it is essential to meet the needs of women during childbirth. However, this study reveals that some women perceived mistreatment by health personnel, who responded negatively or with little empathy to their needs for mobility, food, pain relief, and above all, respect during this vulnerable stage. These unmet needs generate discomfort, unease, and painful situations, which negatively affect their childbirth experience.

*“Giving birth didn't hurt, I didn’t complain during contractions or anything, but I did complain when they were stitching me up inside, because everything was fresh and I begged the doctor not to do it, not to touch me, but he did it anyway. There was a female doctor there, and she was the one who finished stitching me up and checked my tear. They took a long time to stitch me up and then moved me to the ward.”* 28-year-old postpartum woman.

*“I didn't ask for pills for the contractions because it’s a natural process. But when it came to the stitching, I asked, I begged them to give me anesthesia, and they didn’t. They just stitched me up inside. No, no matter how much I begged them to give me anesthesia, they didn’t.”* 28-year-old postpartum woman.

Integration: Physical obstetric violence manifests itself in the lack of support during childbirth, the inability to choose the position in which to give birth, and the failure to allow time for skin-to-skin contact with the baby. These aspects, together with a lack of information about procedures and repetitive gynecological examinations, generate discomfort, unease, and negative emotions in postpartum women, which contributes to the perception of a bad experience during childbirth and the postpartum period.

### Quantitative: Perception of psychological obstetric violence

Among the most frequent manifestations of psychological obstetric violence, 69.1% of postpartum women indicated that they were not informed about giving their informed consent before signing and undergoing any intervention; 53.2% mentioned that the staff who attended their delivery did not identify themselves by name or profession, 48.2% felt that they only focused on their physical health, without considering their emotional needs, and 44.6% were called by diminutive terms (little girl, mommy, etc.). In addition, 32.4% had difficulty expressing their fears and concerns, and 70.5% did not receive information about surgical procedures or interventions before they were performed (Table 4).

**Table 4:** Manifestations of psychological obstetric violence among postpartum women treated at a public hospital in Peru, 2023.

Indicators	Yes		No	
	n	(%)	n	(%)
Personnel attending the birth were not identified	74	(53.2)	65	(46.8)
Comments and expressions such as insults	9	(6.5)	130	(88.7)
Ironic, disparaging, or joking comments about their behavior	11	(7.9)	128	(92.1)
They called her diminutive names (little girl, mommy, skinny girl, chubby girl, etc.)	62	(44.6)	77	(55.4)
Sexist comments and expressions (“women can't stand pain,” “they complain about everything,” “you scream like you’re having your first baby”)	24	(17.3)	115	(82.7)
Their opinion was not taken into account when deciding on matters during childbirth.	34	(24.5)	105	(75.5)
Healthcare professionals used words that they could not understand or found difficult to understand.	38	(27.3)	101	(72.7)
Inadequate explanation of surgical procedures or interventions prior to their performance	41	(29.5)	98	(70.5)
Healthcare professionals focused solely on his health and not on other needs such as his feelings and/or emotions.	67	(48.2)	72	(51.8)
Was not allowed to consume liquids and/or food.	32	(23.0)	107	(77.0)
They did not explain informed consent before signing and performing any intervention.	96	(69.1)	43	(30.9)
She was dissatisfied with the treatment she received from healthcare professionals during her delivery.	37	(26.6)	102	(73.4)
It was difficult for her to ask questions and express her fears and concerns.	45	(32.4)	94	(67.6)

### Qualitative: Meaning and emotions generated by psychological obstetric violence

Interviews revealed the psychological violence perceived by women during childbirth, highlighting the importance of the quality of the treatment. Many reported experiencing mistreatment by health personnel, which caused emotional distress. The lack of information, adequate guidance, negative responses to their needs, and lack of empathy during care caused fear and distress, turning their childbirth experience into an unpleasant one.

*“In an emergency, you are treated badly because they tell you, ‘Lady, stand up, lady, walk.’ So, you are a health professional, you have to be nice. There weren’t many births at that time, I don’t have all night for you, that’s what they told me.”* 21-year-old postpartum woman.

*“Two nurses told me nicely, ‘Come on, you can do it,’ and encouraged me, but this nurse told me, ‘Come on, hurry up and push because that’s why you spread your legs.”* 15-year-old postpartum woman.

Indeed, mistreatment perceived by women during childbirth generates negative emotions that affect the normal process of this stage. This can result in a lack of cooperation, delays in care, increased stress and anxiety, which in turn fosters mistrust and discomfort toward healthcare. In the long term, these experiences can lead to desertion from health facilities.

*“I felt sick, but I still pushed hard and they demanded more of me than I could do. I felt pressured more than anything else […] An obstetrician told me: ‘You’re going to learn on your own, you have to manage by yourself, no one here is going to help you, no one is going to be your employee!'”* 15-year-old postpartum woman.

Integration: Of the two types of obstetric violence considered in the research, psychological violence is the most frequent; and since it is a type of violence that is not visibly evident, it is often difficult to recognize as violence, both by the victims themselves and by the health personnel who perpetrate it; as they tend to normalize situations in which women’s sexual and reproductive rights are violated at this stage, such as not identifying themselves to their patients or using diminutive terms when addressing them. This becomes apparent when the interview is explored in greater depth, with women describing these types of situations as uncomfortable and generating negative feelings.

## DISCUSSION

This study on obstetric violence in a public hospital in Peru has identified several manifestations of this problem, which affects both the physical and emotional health of postpartum women. The demographic characteristics of the women reveal a common profile of young women with secondary education and cohabiting marital status, which could hinder their ability to exercise their rights during the childbirth process. This finding coincides with Huarino and Choque ^13^, who also reported a predominantly young population aged 30 to 45 (57.6%) with secondary education (74.4%), highlighting the relationship between social vulnerability and greater exposure to obstetric violence.

In terms of the perceptions of postpartum women, 25% reported having experienced some form of obstetric violence, a figure that varies according to the international context. For example, in Spain, 34% of women are reported to be affected, while in Ethiopia, the figure reaches 79.7%. Despite regional differences, psychological violence was the most commonly identified form of violence in this study, followed by physical violence. This pattern reflects the systemic nature of obstetric violence, which goes beyond physical procedures and manifests itself more insidiously in a lack of communication, empathy, and respect toward postpartum women.

The psychological manifestations of obstetric violence, such as lack of informed consent before performing any intervention or procedure and the absence of identification of professionals, are consistent with previous studies such as that of Jojoa *et al*. ^23^, which highlight the lack of information provided to women during the birthing process, which affects their ability to make informed decisions and has a negative impact on their emotional well-being.

It is important to add that many of these psychological obstetric violence practices have been normalized in several cultures. An example of this is the use of diminutives such as “*mamita*” (mommy), “*flaquita*” (skinny girl), or “*gordita*” (chubby girl) in Peru, which are perceived by health professionals and users as expressions of “affection” or “esteem.” However, this perception contrasts with the findings of Iglesias’ study ^20^, where such comments, in the Spanish context, are interpreted as disrespectful, paternalistic, or even a form of contempt towards women.

This study also highlights the influence of psychological violence on women’s emotional state, causing feelings of sadness, fear, and distress, as has also been reported in Ecuador ^24^^)^ and Ghana ^25^. Although depression was not measured directly, the emotional effects of obstetric violence found in the participants show a pattern similar to that found in other studies, which show a relationship between mistreatment during childbirth and subsequent emotional disorders.

About half of the postpartum women felt that the health professionals who cared for them did not take their emotional needs and feelings into account, prioritizing routine medical procedures. Similar results were found in Colombia ^23^, where informants reported being limited in freely expressing their emotions, feelings, and concerns, adopting an attitude of submission due to scolding, criticism, and ridicule from health personnel. Likewise, 44.6% of postpartum women perceived psychological violence when they were called by diminutive names; qualitative results showed that they reported feeling uncomfortable and somewhat afraid to communicate their needs to health personnel. In Mexico, 77.8% mentioned that they were criticized with disparaging and ironic comments by healthcare personnel ^26^. However, in Spain ^20^, the figures were lower (5.5%) for women treated in this kind of way.

On the other hand, physical procedures, such as lack of accompaniment, inability to choose the position for delivery, and surgical interventions without prior consent, were also common in this study, similar to findings in a public hospital in Tacna, Peru ^13^, and other countries such as Mexico ^26^^)^ and Chile ^19^. These practices not only affect women’s physical health but also contribute to the deterioration of their emotional health, causing anxiety, stress, and fear. The lack of support during childbirth, for example, has been linked to a greater sense of loneliness and vulnerability, which is completely contradictory to the recommendations of the World Health Organization (WHO), which promote a humanized childbirth that respects the dignity and rights of women ^1^.

Nearly two-thirds of postpartum women reported that they underwent medical and surgical procedures such as episiotomies and episiorrhaphy without prior communication, similar to reports from a public hospital in Tacna, Peru ^13^, where 73.6% said that they were sometimes or never asked for verbal consent for procedures such as vaginal examination (34.8%), and did not receive information about membrane rupture (48.8%), episiotomy (30.8%), or uterine examination (57.6%). In Mexico ^26^, 46.7% did not receive informed consent prior to an episiotomy, and in Chile, 79.2% of women who participated in the study reported that procedures were performed without proper explanation or informed consent ^19^.

Finally, experiences of obstetric violence related to repetitive vaginal touching and the unconsented presence of other people during the care process reflect a lack of respect for the privacy and autonomy of postpartum women. These situations of distress, discomfort, and frustration, in which women feel powerless in the face of a lack of information and the repetition of unsolicited procedures, are consistent with studies from other countries, highlighting a global problem ^27^^-^^29^.

This study highlights the gap in compliance with WHO guidelines and in the implementation of a health system that guarantees humanized childbirth. The reported practices continue to fall short of ensuring respectful and dignified treatment for women, underscoring the urgent need to strengthen education and empowerment of postpartum women regarding their reproductive rights ^30^. Although the study has limitations, such as possible memory bias among participants and sample size, its mixed approach provides a more comprehensive and detailed view of obstetric violence, contributing to the understanding of this phenomenon and reflection on the actions needed to eradicate it.

In conclusion, postpartum women perceive obstetric violence both psychologically and physically, and it is linked to the quality of treatment received from healthcare professionals and the procedures performed during childbirth. Psychological violence is mainly experienced through a lack of information, poor communication, and a lack of empathy on the part of healthcare personnel. On the other hand, physical violence manifests itself in the performance of procedures without consent or without adequate explanation, invasive and painful maneuvers, restriction of movement, and repetitive procedures, which negatively impact women’s perception of safety and well-being during childbirth.

Qualitative narratives reveal that postpartum women feel that the birth process is experienced in a depersonalized manner, reinforcing a sense of dehumanization by healthcare professionals. In addition, many women have come to normalize certain practices of obstetric violence, reflecting a process of desensitization to such treatment, viewing it as inevitable within the healthcare process. Additional research is needed to contribute to the design of public policies aimed at preventing and addressing obstetric violence in the country, with the goal of improving the quality of care and ensuring more respectful and humane care for women during childbirth.
