# Supplementary material for: Perception and experience of obstetric violence in postpartum women at a public hospital in Peru: a mixed study
Source: Rev Peru Med Exp Salud Publica. 2025 Mar 11;42(1):54–62. doi: 10.17843/rpmesp.2025.421.14281 (PMC12176021; doi:10.17843/rpmesp.2025.421.14281)
Supplement: Translation

# Translation: Percepción y experiencia de violencia obstétrica en puérperas de un hospital público en Perú: un estudio mixto

**Authors:** Paola M. Marcos-Garces, Stefanny M. Moran-Ruiz, Yuly R. Santos-Rosales, Miriam Y. Correa-Lopez

**Affiliations:** 1 Escuela Profesional de Obstetricia, Facultad de Medicina, Universidad Nacional Mayor de San Marcos, Lima, Perú.

**Keywords:** Violencia obstétrica, Violencia contra la mujer, Violencia de género, Parto humanizado, Parto obstétrico

## Abstract

**Objetivos.:**

Determinar la percepción y experiencia de violencia obstétrica durante el parto en puérperas de un hospital público en Perú.

**Materiales y métodos.:**

Estudio mixto, descriptivo y transversal en la fase cuantitativa y con diseño fenomenológico en la fase cualitativa. La población fue de 444 puérperas, con una muestra conformada por 139 puérperas de parto vaginal (encuestas) y 21 puérperas (entrevistas semiestructuradas). Se realizó durante los meses de abril a diciembre del 2023.

**Resultados.:**

El 25,2% de las mujeres encuestadas percibieron violencia obstetrica durante el parto y el puerperio; sin embargo, al preguntar por manifestaciones concretas de violencia obstétrica el 100% de las puérperas reportaron haber experimentado alguna forma de violencia física y el 97,8% respondió que experimentó alguna manifestación de violencia psicológica obstétrica, y todas las participantes indicaron haber sufrido al menos una forma de violencia obstétrica. Sobre la violencia psicológica, del total de puérperas, 69,1% percibieron que no les explicaron sobre el consentimiento informado antes de firmar y realizar alguna intervención, 53,2% manifestó que el personal que la atendió el parto no se identificó con su nombre ni con su profesión. Sobre la violencia obstétrica física; el 96,4% no tuvieron a una persona de confianza durante el parto, 91,4% no tuvo la opción de elegir la posición para dar a luz (horizontal o vertical), en el 76,3% no se respetó la hora de contacto piel a piel con su bebé. La violencia obstétrica se expresa en sentimientos de temor, angustia, ansiedad, frustración y soledad, que pone en riesgo la salud materno neonatal.

**Conclusiones.:**

Las puérperas perciben la violencia obstétrica a nivel psicológico por la calidad del trato de los profesionales de la salud y a nivel físico por las prácticas realizadas durante su parto, las cuales afectan negativamente sus experiencias generando en ellas un impacto emocional.

## INTRODUCCIÓN

El parto es un proceso fisiológico que culmina con la expulsión del producto concebido y sus anexos ^1^. Sin embargo, la medicalización y el uso de tecnología han transformado el parto humanizado al priorizar intervenciones médicas, reduciendo la autonomía de la mujer y despersonalizando la experiencia del nacimiento ^2^^,^^3^. Esto ha generado una tensión entre el respeto por los procesos naturales y la intervención médica excesiva, afectando la experiencia del parto. La Organización Mundial de la Salud (OMS) reconoce el derecho de la mujer a recibir atención digna, libre de violencia, pero se reportan hechos que generan desconfianza y rechazo hacia los servicios de salud ^4^. Por tanto, es esencial brindar atención de calidad, respetando la autonomía de la mujer, a través de prácticas como el acompañamiento de parto por alguien de su elección, la comunicación efectiva entre médico y paciente, y la promoción de la movilidad, elección de posiciones, acceso a líquidos y alivio del dolor ^1^. Estas acciones están alineadas con el tercer Objetivo de Desarrollo Sostenible, que busca promover la salud y el bienestar en todas las etapas de la vida ^5^^,^^6^ .

A nivel mundial, no existe un consenso sobre la definición de violencia obstétrica ^7^^-^^10^. En el Perú, esta forma de violencia es reconocida como una modalidad de violencia de género ^11^. Según el “Plan Nacional Contra la Violencia de Género 2016-2021”, se entiende como “todos los actos de violencia por parte del personal de salud relacionados con los procesos reproductivos, que se expresan en un trato deshumanizador, abuso de medicalización y patologización de los procesos naturales” ^12^^,^^13^.

La violencia obstétrica se manifiesta en diversas situaciones, como la no aplicación del consentimiento informado por parte del personal de salud (73,6%), el desistimiento de la opinión y decisión de la paciente, la falta de información sobre el tacto vaginal (34,8%) ^14^, la ausencia de identificación del personal de salud por su nombre (32,8%), la negación del acompañamiento durante el parto (90,4%), la administración improcedente de medicación (99,6%) ^13^ o la realización de procedimientos por parte de estudiantes sin el consentimiento de la paciente ^3^^,^^12^^,^^13^. Estas prácticas reflejan la violación de los derechos reproductivos de las mujeres, generando desconfianza hacia los servicios de salud y perpetuando la violencia estructural en la atención obstétrica ^15^^,^^16^.

La violencia obstétrica es un problema que requiere atención urgente debido a sus efectos negativos ^17^ en la vida de las mujeres, en el acceso oportuno a la atención sanitaria y por vulnerar sus derechos reproductivos. En este contexto, el objetivo de este estudio fue determinar la percepción de violencia obstétrica durante el parto en puérperas de un hospital público del Perú en 2023.

**MENSAJE CLAVE**

Motivación para realizar el estudio. Existen brechas de atención humanizada durante el parto, donde se evidencia la violencia obstétrica, siendo importante conocer las percepciones de las puérperas, sobre este tipo de violencia.

Principales hallazgos. El 25,2% refiere haber percibido violencia obstétrica; sin embargo, todas las participante experimentaron alguna manifestación de violencia obstetrica como: que no recibieron explicación sobre el consentimiento informado para realizarles procedimientos, el personal de salud no se identifica y no tuvieron la opción de elegir la posición y acompañamiento durante el parto.

Implicancias. Urge empoderar a las gestantes sobre la importancia de ejercer sus derechos reproductivos, identificando la violencia obstétrica; a fin de implementar recomendaciones y condiciones para un parto humanizado.

## MATERIALES Y MÉTODOS

### Diseño del estudio y muestra

Estudio de enfoque mixto ^18^ con diseño de triangulación concurrente, lo que permitió la recolección y análisis de los datos cuantitativos y cualitativos en simultáneo, para ser integrados posteriormente en la fase de interpretación. En cuanto al enfoque cuantitativo, se utilizó un diseño descriptivo transversal, mientras que el enfoque cualitativo siguió un diseño fenomenológico, centrado en describir y comprender las experiencias de las participantes en relación con la violencia obstétrica experimentada; este enfoque permitió obtener una visión integral de las percepciones y manifestaciones de la violencia obstétrica, contrastando las evidencias numéricas con los relatos subjetivos de las puérperas.

La muestra estuvo conformada por 139 y se utilizó la fórmula de Fisher para extraer una muestra representativa. N: universo o población 444, p: probabilidad a favor 0,5, q: probabilidad en contra 0,5, n: tamaño de la muestra 139, e: error de estimación 0,03, alfa: coeficiente de nivel de confianza 1,960.

Para el enfoque cuantitativo, la estimada del Hospital Nacional Daniel Alcides Carrión fue de 444 puérperas (según registro del año 2022) y la muestra fue de 139 puérperas determinadas por muestreo aleatorio simple y para el enfoque cualitativo; las informantes fueron 21 puérperas, seleccionadas por criterio de saturación teórica.

Los criterios de selección de la población fueron: todas las puérperas de parto vaginal del Hospital Nacional Daniel Alcides Carrión en la región del Callao en Perú, atendidas entre abril y diciembre del 2023 (cuantitativa) y para el enfoque cualitativo se incluyeron a las puérperas que respondieron sí a las preguntas de violencia obstétrica. Asimismo, para ambos enfoques se excluyeron a puérperas de nacionalidad extranjera, idioma distinto al castellano, habilidades especiales o internadas en la Unidad de Cuidados Intensivos y quienes no firmaron consentimiento y/o asentimiento informado.

### Variables, instrumento y recolección de la información

Para el enfoque cuantitativo, la violencia obstetrica (VO) fue evaluada de dos formas, una a través de una pregunta directa para explorar si perciben haber sufrido violencia obstetrica y la otra forma sobre las experiencias concretas de VO en dos dimensiones violencia física y psicológica. Se consideró que la gestante experimentó VO cuando presentó por lo menos alguna manifestación física o psicológica ^19^, en esta definición y clasificación se tomó en cuenta a la OMS ^2^, Defensoría del Pueblo ^11^ y Plan Nacional de Violencia de Género ^12^.

Se categorizó como violencia psicológica cuando se encontró una respuesta positiva a las siguientes manifestaciones: expresiones denigrantes, discriminatorias, irónicas y descalificadoras; regaños por expresar dolor, omisión del consentimiento informado, entre otras ^3^^,^^12^. Mientras que para la violencia física se consideró la respuesta positiva a alguna de las siguientes manifestaciones: tacto vaginal sin justificación razonable, maniobras de Kristeller o Hamilton, medicalización durante el parto, ruptura artificial de membranas, pinzamiento precoz del cordón umbilical, denegación de la movilidad injustificada durante el parto, imposibilidad de elegir la posición de parto, presencia de terceros ajenos a la sala de parto sin el consentimiento de la madre, entre otros) ^3^^,^^20^. Por su parte para la fase cualitativa la categoría principal fue la percepción de VO y se consideraron dos subcategorias: Significado y emociones generada por la violencia obstétrica física y significado y emociones generadas por la violencia obstétrica psicológica.

En el enfoque cuantitativo la técnica fue la encuesta y el instrumento fue adaptado del Informe de Adjuntía N°023-2020-DP/ADM ^12^, de la encuesta del artículo Violencia obstétrica en Chile: percepción de las mujeres y diferencias entre centros de salud ^19^, de la encuesta del artículo ¿Violencia obstétrica en España, realidad o mito? 17 000 mujeres opinan ^20^. El cuestionario consta de 38 preguntas estructuradas en tres bloques; fue validado por juicio de expertos, cuenta con validez de V de Aiken (0,96) y confiabilidad de 0,77 (Kunder Richardson). El bloque I sobre violencia psicológica con 16 preguntas, bloque II sobre violencia física 17 preguntas y bloque III sobre información de violencia obstétrica 5 preguntas; las puérperas fueron seleccionadas mediante un muestreo aleatorio simple (sorteo).

En el enfoque cualitativo la técnica fue la entrevista y el instrumento fue una guía de entrevista semiestructurada con 18 preguntas dividida en 2 bloques, I sobre violencia psicológica con 7 preguntas y II sobre violencia física con 11 preguntas. El contenido de la guía de entrevista fue validado por juicio de expertos, quienes revisaron el contenido para asegurar la claridad y la pertinencia de las preguntas (validez V AIKEN de 0,90), las entrevistas fueron realizadas en espacios sin distracciones y resguardando la confidencialidad, con un promedio de 20 minutos de duración; fueron grabadas previo consentimiento y asentimiento informado para menores de edad independizados, para luego transcribirlas y analizarlas.

### Análisis

Para el análisis cuantitativo se procesaron y analizaron los datos con el programa estadístico SPSS versión v.29. Se calculó la frecuencia y porcentaje de las variables estudiadas. Para el análisis cualitativo; se organizaron los datos mediante la transcripción de las entrevistas; luego se procedió a la codificación; donde se identificó, etiquetó y agrupó, palabras y segmentos de datos relevantes para el estudio, con el fin de explorar ideas o patrones repetitivos en las entrevistadas. Luego se procedió a la categorización, donde se identificó las similitudes y diferencias entre las unidades codificadas, a fin de agruparlas en categorías temáticas, que para efectos del estudio fueron los distintos tipos de violencia obstétrica que experimentaron las participantes, los mismos que fueron consignados en una matriz de categorización; para finalmente proceder con la interpretación de los resultados.

Para la integración de resultados cuantitativos y cualitativos, se aplicó el método de triangulación concurrente o convergente ^18^, el cual se basó en la recopilación y análisis de los datos cuantitativos y cualitativos de manera simultánea, para luego ser integrados en base a puntos de convergencia y complementariedad en los hallazgos, siendo estos puntos convergentes la violencia obstétrica física y la violencia obstétrica psicológica a nivel de percepciones y de experiencias, lo cual contribuye a obtener una interpretación conjunta y completa del fenómeno estudiado (violencia obstétrica) Figura 1.

**Figura 1:** Esquema de triangulación de datos cuantitativos y cualitativos para violencia obstétrica psicológica.

### Consideraciones éticas

El estudio tuvo la aprobación del Comité de ética del Hospital Nacional Daniel Alcides Carrión (N°015 - 2023). Previo al recojo de datos, las participantes conocieron la finalidad del estudio, la participación voluntaria a través del consentimiento y asentimiento informado para menores de edad independizadas y aquellas que no, se les solicitó consentimiento informado a sus padres o apoderados ^21^. Se aseguró la confidencialidad de los datos a través del anonimato y se expresó el uso exclusivo para fines de investigación.

## RESULTADOS

Para efectos de interpretación se consideró como dimensiones para el enfoque cuantitativo a la caracterización de la población, violencia obstétrica física y psicológica; mientras que para el enfoque cualitativo las categorías fueron, la experiencia de parto y puerperio, significado y emociones generadas por la violencia obstétrica física; significado y emociones generadas por la violencia obstétrica psicológica.

### Cuantitativo: Caracterización de la población estudiada

La mayoría de las puérperas (72%) tienen entre 20 y 34 años, con un 18% entre 14 y 19 años. El 69% tiene estado civil de conviviente, el 70,5% tiene educación secundaria y el 71,2% son amas de casa (Tabla 1). En relación con la violencia obstétrica, esta se abordó inicialmente mediante una pregunta directa, encontrando que el 25,2% de las mujeres encuestadas percibieron haber sufrido algún tipo de violencia obstétrica durante el parto y el puerperio. No obstante, al preguntar por las manifestaciones concretas de VO el 100% de mujeres reportó haber experimentado algún tipo de VO, un 100% violencia física obstetrica y un 97,8% violencia psicológica. Estos resultados indican que, aunque la violencia obstétrica es ampliamente experimentada, solo una cuarta parte de las puérperas la reconocen como tal (Tabla 2).

**Tabla 1:** Características generales de las puérperas que participaron en el estudio de un hospital público de Lima, Perú 2023.

Características generales (n=139)	n	(%)
Edad		
14 -19 años	25	(18,0)
20-34 años	100	(72,0)
>34 años	14	(10,0)
Estado civil		
Soltera	39	(28,1)
Casada	4	(2,9)
Conviviente	96	(69,0)
Grado de instrucción		
Primaria	5	(3,6)
Secundaria	98	(70,5)
Superior técnico	28	(20,1)
Superior universitario	8	(5,8)
Ocupación		
Ama de casa	99	(71,2)
Estudiante	9	(6,5)
Trabajadora dependiente	7	(5,0)
Trabajadora independiente	24	(17,3)

**Tabla 2:** Proporción de puérperas que experimentó violencia obstétrica en un hospital público del Perú, 2023

Característica (n=139)	n	%
Experimentó al menos una manifestación de violencia obstétrica (física o psicológica)	139	100
Experimentó violencia obstétrica física	139	100
Experimentó violencia obstétrica psicológica	136	97,8
Experimentó ambos tipos de violencia obstétrica	136	97,8

### Cualitativo: Experiencias durante el parto y puerperio

En el componente cualitativo, un hallazgo común entre las entrevistadas fue la diversidad de experiencias durante el parto. Para la mayoría, fue una experiencia agradable por la emoción de tener a sus hijos, mientras que para otras resultó incómoda y dolorosa, ya sea por la naturaleza fisiológica del parto o por situaciones percibidas como malos tratos por parte del personal de salud. Entre estas, se destacó la falta de información y orientación adecuada sobre los procedimientos a seguir, lo que provocó demoras en la atención y desplazamientos innecesarios entre establecimientos, aumentando el riesgo de complicaciones y de partos fuera del lugar adecuado o sin el personal médico idóneo.

*“Estaba en 2 de dilatación me dijeron que regrese dentro de 5 horas […] cuando llegué a las 10 de la noche al centro de salud me tocaron de nuevo y me dijeron que estaba en 5, pero me dijeron que no me podían atender aquí en la posta, que me vaya al hospital […] Entonces me negaron la atención, tuve que venir por mi cuenta a las 11pm no había taxi, llegué con 8 de dilatación. Cuando llegue a Emergencias de Carrión solicite un silla y me dijeron no hay sillas, me hicieron caminar y cuando llegue le dije que ya estaba dilatando y me pidieron mi referencia le explique que no tenía, porque en la posta me dijeron vete al hospital ahí te van a atender de todas maneras, me aceptaron me hicieron sentar y me hicieron tacto y estaba en 10 […] todo fue muy rápido me trasladaron de cama en cama, yo estaba aguantando los pujos y yo gritaba del dolor y me decían pero no grite, cuando llegue aquí pase a dos camillas más me atendieron con la misma bata que yo tenía porque ya estaba en 10, me decían cierre su pierna porque ya se le va salir […]”.* Puérpera de 21 años

Integración: Las mujeres que han experimentado violencia obstétrica durante el parto y puerperio suelen presentar características diferentes a aquellas que no la han vivido, lo que podría convertirse en un factor de riesgo para desarrollarla, dependiendo del contexto y las condiciones sociales, culturales y económicas. Además, es importante considerar que el significado de la violencia obstétrica varía según las características, condiciones y contextos en los que se experimenta, lo que influye en la percepción y el impacto de esta experiencia.

### Cuantitativo: Percepciones sobre la violencia obstétrica física

Entre las manifestaciones más frecuentes de violencia obstétrica, el 96,4% de las mujeres indicó que no se les permitió la presencia de una persona de confianza durante todo el proceso de parto, el 91,4% no tuvo la opción de elegir la posición para dar a luz, y el 76,3% señaló que no se respetó la hora de contacto piel a piel con su bebé. Además, el 71,2% experimentó un inicio tardío de la lactancia materna, el 63,6% tuvo una ruptura artificial de membranas sin previa comunicación, y el 47,5% sufrió tactos vaginales repetitivos por parte de más de una persona. También, el 36,7% permitió la presencia de otras personas durante su atención sin consentimiento, el 32,4% tuvo un pinzamiento precoz del cordón umbilical, y el 23% recibió medicamentos sin su conocimiento ni explicación previa. Otras manifestaciones de violencia obstétrica fueron reportadas por menos del 20% de las participantes (Tabla 3).

**Tabla 3:** Manifestaciones de violencia obstetrica física en puérperas atendidas en un hospital público del Perú, 2023.

Indicadores	Sí		No	
	n	(%)	n	(%)
Tacto vaginal repetitivo por más de una persona en un determinado momento	66	(47,5)	73	(52,5)
Maniobras como Kristeller, Maniobra de Hamilton, etc.	22	(15,8)	117	(84,2)
Intervenciones quirúrgicas (Episiotomía, episiorrafia)	98	(70,5)	41	(29,5)
Administración de medicamentos sin tener conocimiento o sin darle alguna explicación de su importancia o de los efectos de este (oxitocina, hioscina, dimenhidrinato, etc.)	32	(23,0)	107	(77,0)
Ruptura artificial de membranas	88	(63,6)	51	(36,7)
Pinzamiento precoz del cordón umbilical	45	(32,4)	94	(67,6)
Impedimento de la movilidad o posición más cómoda antes del parto	25	(18,0)	114	(82,0)
No tener la opción de elegir la posición para dar a luz (horizontal o vertical)	127	(91,4)	12	(8,6)
No permitir la presencia de una persona de confianza y elección durante todo el proceso del parto	134	(96,4)	5	(3,6)
No colocar al bebé inmediatamente en contacto piel a pesar de que nació sano	5	(3,6)	134	(96,4)
No se respetó la hora del contacto piel a piel madre-hijo.	106	(76,3)	33	(23,7)
Inicio tardío de lactancia materna	99	(71,2)	40	(28,8)
Permitir la presencia de otras personas durante la evaluación o atención sin consentimiento (alumnos en formación, internos, etc.)	51	(36,7)	88	(63,3)
Después del parto el personal introdujo toda su mano para sacar los restos de placenta	96	(69,1)	43	(30,9)
No se administró medicamentos para disminuir el dolor cuando realizaron una revisión con instrumentos para evaluar el útero porque quedaron restos de placenta	104	(74,8)	35	(25,2)
Introdujeron un dedo en el ano sin autorización o explicación previa	6	(4,3)	133	(95,7)
Tomaron fotografía del proceso del parto sin consentimiento	7	(5,0)	132	(95,0)

### Cualitativo: Significado y emociones generada por la violencia obstétrica física

En los resultados cualitativos relacionados con la violencia obstétrica física, se destacó la importancia de los procedimientos utilizados para monitorizar la evolución de las gestantes durante el parto. Muchas mujeres expresaron sentirse incómodas, ya que, aunque comprenden que ciertos procedimientos son necesarios, no fueron informadas o consultadas para obtener su consentimiento. Además, la repetición de estos procedimientos sin explicación, y la intervención de varios profesionales de salud, a pesar de sus molestias e incomodidad, les generó una sensación de vulnerabilidad y afectación emocional.

*“Sí, les dije que me incomodaba y bueno, ellas me dijeron, pero te hemos puesto anestesia (analgesia) no te puede doler; […] pues eso también incómoda bastante porque cada cinco minutos creo me metían la mano 5 minutos venía otro, venía otro y como que te tocaban y te jalaban no sé qué y más se venían los dolores y uno le decía que no y seguían. Él solo lo hacía, pero de ahí se iba y venía otro doctor y te metía la mano”.* Puérpera de 16 años.

Cubrir las necesidades de los pacientes en los establecimientos de salud es un indicador clave de calidad, vinculado al nivel de satisfacción y bienestar de las usuarias. En este sentido, es esencial satisfacer las necesidades de las mujeres durante el parto. Sin embargo, este estudio revela que algunas mujeres perciben malos tratos por parte del personal de salud, quienes respondieron de manera negativa o poco empática ante sus necesidades de movilización, alimentación, alivio del dolor y sobre todo, respeto en esta etapa vulnerable. Estas necesidades insatisfechas generan malestar, incomodidad y situaciones dolorosas, lo que afecta negativamente su experiencia del parto.

*“No me dolía el parto, no me quejé en las contracciones ni nada, pero me quejé a la hora de coserme por dentro, por lo que estaba todo fresco y yo le supliqué al doctor que no lo hiciera que no me tocara, pero igual lo hizo. Justo estaba una doctora y fue la que me terminó de coser y ver todo el tema de mi desgarro, pues de ahí se demoraron en coserme y me pasaron a piso”.* Puérpera de 28 años.

*“Para las contracciones no pedí pastillas, porque es un proceso natural. Pero para el tema que me cosieron si solicité, pedí, imploré que me pusieran anestesia y no me pusieron. Me cosieron por dentro no más. No, por más que supliqué que me pusieran anestesia, no me pusieron”.* Puérpera de 28 años.

Integración: La violencia obstétrica física se manifiesta en la falta de acompañamiento durante el parto, la imposibilidad de elegir la posición para dar a luz y la no consideración del tiempo para el contacto piel a piel con el bebé. Estos aspectos, junto con la falta de información sobre los procedimientos y los exámenes ginecológicos repetitivos; generan incomodidad, malestar y emociones negativas en las puérperas lo cual contribuye a la percepción de una mala experiencia durante el parto y puerperio.

### Cuantitativo: Percepción de la violencia obstétrica de tipo psicológico

Entre las manifestaciones más frecuentes de violencia obstétrica de tipo psicológica, el 69,1% de las puérperas indicó que no se les explicó sobre el consentimiento informado antes de firmar y realizar alguna intervención. El 53,2% mencionó que el personal que atendió su parto no se identificó con su nombre ni profesión, el 48,2% sintió que solo se enfocaron en su salud física, sin considerar sus necesidades emocionales, y el 44,6% fue llamada con diminutivos (hijita, mamita, etc.). Además, el 32,4% tuvo dificultad para expresar sus miedos e inquietudes, y el 70,5% no recibió información sobre los procedimientos o intervenciones quirúrgicas antes de realizarlos (Tabla 4).

**Tabla 4:** Manifestaciones de violencia obstetrica psicológica de las puérperas atendidas en un hospital público del Perú, 2023.

Indicadores	Sí		No	
	n	(%)	n	(%)
No identificación del personal que atiende el parto	74	(53,2)	65	(46,8)
Comentarios y expresiones como insultos	9	(6,5)	130	(88,7)
Comentarios irónicos, descalificadores o en tono de chiste acerca de su comportamiento	11	(7,9)	128	(92,1)
La llamaron con diminutivos (hijita, mamita, flaquita, gordita, etc)	62	(44,6)	77	(55,4)
Comentarios y expresiones sexistas (“las mujeres no aguantan el dolor”, “de todo se quejan”, “gritas como si fueras primeriza”)	24	(17,3)	115	(82,7)
Su opinión no fue tomada en cuenta para decidir situaciones durante la atención de parto	34	(24,5)	105	(75,5)
Los profesionales de la salud emplearon palabras que no pudo o le fue difícil de entender	38	(27,3)	101	(72,7)
Deficiente explicación sobre los procedimientos o intervenciones quirúrgicas antes de su realización	41	(29,5)	98	(70,5)
Los profesionales de la salud solo se enfocaron en su estado de salud y no en las otras necesidades como sus sentimientos y/o emociones	67	(48,2)	72	(51,8)
No se le permitió consumir líquidos y/o alimentos	32	(23,0)	107	(77,0)
No le explicaron sobre el consentimiento informado antes de firmar y realizar alguna intervención.	96	(69,1)	43	(30,9)
No se sintió satisfecha con el trato brindado por los profesionales de la salud durante la atención de su parto	37	(26,6)	102	(73,4)
Le fue difícil preguntar, manifestar sus miedos e inquietudes	45	(32,4)	94	(67,6)

### Cualitativo: Significado y emociones generada por la violencia obstétrica psicológica

Las entrevistas revelaron la violencia psicológica percibida por las mujeres durante el parto, destacando la importancia de la calidad del trato recibido. Muchas expresaron haber experimentado malos tratos por parte del personal de salud, lo que generó malestar emocional. La falta de información, orientación adecuada, respuestas negativas a sus necesidades y la falta de empatía durante la atención provocaron temor y angustia, convirtiendo su experiencia de parto en algo desagradable.

*“En emergencia un mal trato porque te dicen señora párese, señora camine; entonces eres un profesional de la salud debes ser amable, no había muchos partos en ese momento, no tengo toda la noche para usted, así me dijeron”.* Puérpera de 21 años

*“Como dos enfermeras me dijeron de bonita manera, me decían vamos, tú puedes, me dieron ánimos, pero esta enfermera me decía vamos, apúrate que tienes que pujar porque para eso has abierto las piernas”.* Puérpera de 15 años

Efectivamente, los malos tratos percibidos por las mujeres durante el parto generan emociones negativas que afectan el proceso normal de esta etapa. Esto puede resultar en falta de colaboración, demoras en la atención, mayor estrés y ansiedad, lo que a su vez fomenta desconfianza y malestar hacia la atención recibida. A largo plazo, estas experiencias pueden llevar a la deserción de los establecimientos de salud.

*“Me sentí mal, todavía hice fuerza y me exigían más de lo que yo no podía hacer. Me sentí presionada más que todo […] Una obstetra me dijo: ¡Vas a aprender tú solita, tienes que valerte por ti sola, acá nadie te va a ayudar nadie va a ser tu empleado!”.* Puérpera de 15 años

Integración: De los dos tipos de violencia obstétrica considerados en la investigación, la violencia psicológica es la más frecuente; y tratándose de un tipo de violencia que no se evidencia visiblemente, muchas veces aún es difícil reconocerla como violencia tanto por las mismas víctimas, así como por parte del personal de salud que la ejerce; ya que suelen normalizar situaciones en las que se vulnera los derechos sexuales y reproductivos de las mujeres en esta etapa, como el hecho de no identificarse ante sus pacientes o hacer uso de diminutivos para dirigirse a ellas; lo cual se manifiesta cuando se logra profundizar en una entrevista, donde señalan a este tipo de situaciones como incómodas y que generan sentimientos negativos en estas mujeres.

## DISCUSIÓN

El presente estudio sobre violencia obstétrica en un hospital público del Perú ha permitido identificar las diversas manifestaciones de esta problemática, que afecta tanto a la salud física como emocional de las mujeres puérperas. Las características demográficas de las mujeres atendidas revelan un perfil común de jóvenes, con nivel educativo secundario y de estado civil convivientes, lo que podría dificultar su capacidad para ejercer sus derechos durante el proceso de parto; este hallazgo coincide con Huarino y Choque ^13^, que también reportaron una población predominantemente joven de 30 a 45 años (57,6%) y con escolaridad secundaria (74,4%), lo que resalta la relación entre la vulnerabilidad social y la mayor exposición a la violencia obstétrica.

En términos de las percepciones de las puérperas, el 25% manifestó haber experimentado algún tipo de violencia obstétrica, cifra que varía según el contexto internacional, por ejemplo, en España se reporta un 34% ^20^^)^ de mujeres afectadas, mientras que en Etiopía la cifra alcanza el 79,7% ^22^. A pesar de las diferencias regionales, la violencia psicológica fue la más comúnmente identificada en este estudio, seguido por la violencia física. Este patrón refleja la naturaleza sistémica de la violencia obstétrica, la cual va más allá de los procedimientos físicos y se manifiesta de forma más insidiosa en la falta de comunicación, empatía y respeto hacia las puérperas.

Las manifestaciones psicológicas de violencia obstétrica, tales como la falta de consentimiento informado antes de realizar alguna intervención o procedimiento y la ausencia de identificación de los profesionales, concuerdan con estudios previos como el de Jojoa *et al*. ^(^^23^, que resaltan la falta de información proporcionada a las mujeres durante el proceso de parto, lo que afecta su capacidad para tomar decisiones informadas y ejerce un impacto negativo sobre su bienestar emocional.

Es importante añadir que muchas de estas prácticas de violencia obstétrica psicológica han sido normalizadas en diversas culturas. Un ejemplo de ello es el uso de diminutivos como “mamita”, “flaquita” o “gordita” en Perú, los cuales son percibidos por los profesionales de la salud y las usuarias como expresiones de “cariño” o “estima”. Sin embargo, esta percepción contrasta con los hallazgos del estudio de Iglesias ^20^, donde este tipo de comentarios, en el contexto de España, son interpretados como una falta de respeto, un acto de paternalismo o incluso una forma de menosprecio hacia las mujeres.

Este estudio también destaca la influencia de la violencia psicológica en el estado emocional de las mujeres, generando sentimientos de tristeza, miedo y angustia, como también se ha reportado en Ecuador ^24^^)^ y Ghana ^25^. Aunque no se midió la depresión de forma directa, los efectos emocionales de la violencia obstétrica observados en las participantes señalan un patrón similar al de otras investigaciones, que evidencian la relación entre los malos tratos durante el parto y los trastornos emocionales subsecuentes.

Cerca de la mitad de las puérperas percibieron que los profesionales de la salud que las atendieron no tomaron en cuenta sus necesidades emocionales y sentimientos, priorizando procedimientos médicos de rutina; resultados similares se encontraron en Colombia ^23^, donde sus informantes manifestaron haber sido limitadas a expresar con libertad sus emociones, sentimientos e inquietudes, adoptando una actitud de sumisión, debido a regaños, críticas y burlas por parte del personal de salud. Asimismo, el 44,6% de puérperas percibieron violencia psicológica cuando se les llamaba con diminutivos; en los resultados cualitativos reportaron sentirse incómodas y con cierto temor de comunicar sus necesidades al personal de salud. También, en México el 77,8% mencionan que fueron criticadas con comentarios descalificadores e irónicos por parte del personal de salud ^26^. No obstante, en España ^20^, las cifras fueron menores (5,5%) de mujeres tratadas con este tipo de diminutivos.

Por otro lado, los procedimientos físicos, como la falta de acompañamiento, la imposibilidad de elegir la posición para el parto, y la realización de intervenciones quirúrgicas sin consentimiento previo, fueron también comunes en este estudio, similar a los hallazgos en un Hospital público de Tacna en Perú ^13^, y otros países como México ^26^ y Chile ^19^. Estas prácticas no solo afectan la salud física de las mujeres, sino que también contribuyen a la alteración de su salud emocional, generando ansiedad, estrés y temor. La falta de acompañamiento durante el parto, por ejemplo, se ha vinculado con una mayor sensación de soledad y vulnerabilidad, lo cual es completamente contradictorio con las recomendaciones de la Organización Mundial de la Salud (OMS), que promueven un parto humanizado que respete la dignidad y los derechos de las mujeres ^1^.

Cerca de las dos terceras partes de las puérperas manifestaron que les realizaron procedimientos médicos y quirúrgicos como episiotomías y episiorrafias sin comunicación previa, similar a lo reportado en un Hospital público de Tacna en Perú ^13^, donde al 73,6 manifestaron que a veces o nunca les pidieron consentimiento verbal para procedimientos, como tacto vaginal (34,8%), no recibieron información sobre ruptura de membranas (48,8%), episiotomía (30,8%) o revisión uterina (57,6%). En México ^26^, el 46,7% no recibieron un consentimiento informado previo a la realización de una episiotomía y en Chile el 79,2% de las mujeres que participaron del estudio manifestaron que se les realizó procedimientos sin una debida explicación o sin un consentimiento informado ^19^.

Finalmente, las experiencias de violencia obstétrica relacionadas con el tacto vaginal repetitivo y la presencia no consentida de otras personas durante el proceso de atención reflejan una falta de respeto por la privacidad y autonomía de las puérperas. Estas situaciones de malestar, incomodidad y frustración, en las cuales las mujeres se sienten impotentes ante la falta de información y la reiteración de procedimientos no solicitados, son coincidentes con estudios de otros países, lo que pone en evidencia una problemática de alcance global ^27^^-^^29^.

Este estudio resalta la brecha en el cumplimiento de las normativas de la OMS y en la implementación de un sistema de salud que garantice el parto humanizado. Las prácticas observadas siguen estando lejos de garantizar un trato respetuoso y digno para las mujeres, lo que subraya la necesidad urgente de fortalecer la educación y empoderamiento de las puérperas sobre sus derechos reproductivos ^30^. Aunque el estudio tiene limitaciones, como los posibles sesgos de memoria de las participantes y el tamaño de la muestra, su enfoque mixto permite ofrecer una visión más completa y detallada de la violencia obstétrica, contribuyendo a la comprensión de este fenómeno y a la reflexión sobre las acciones necesarias para erradicarla.

En conclusión, las puérperas perciben la violencia obstétrica tanto en el ámbito psicológico como físico, y están vinculadas a la calidad del trato recibido por los profesionales de salud y los procedimientos realizados durante su atención en el parto. La violencia psicológica se experimenta principalmente a través de la falta de información, comunicación deficiente y la falta de empatía por parte del personal de salud. Por otro lado, la violencia física se manifiesta en la realización de procedimientos sin consentimiento o sin una explicación adecuada, maniobras invasivas y dolorosas, limitación del movimiento y procedimientos repetitivos, lo que impacta negativamente en la percepción de seguridad y bienestar de las mujeres durante el parto.

Las narrativas cualitativas revelan que las puérperas sienten que el proceso de parto se vive de manera despersonalizada, lo que refuerza una experiencia de deshumanización por parte de los profesionales de salud. Además, muchas mujeres han llegado a normalizar ciertas prácticas de violencia obstétrica, lo que refleja un proceso de desensibilización ante estos tratos, viéndolos como algo inevitable dentro del proceso de atención en salud. Es necesario realizar investigaciones adicionales, que puedan contribuir al diseño de políticas públicas orientadas a la prevención y atención de la violencia obstétrica en el país, con el objetivo de mejorar la calidad del cuidado y garantizar una atención más respetuosa y humana para las mujeres durante el parto.
